# The Archives of Pediatrics

**Published:** 1896-01

**Authors:** 


### The Archives of Pediatrics

Has been purchased by Mr. E. B. Treat, the publisher, 5 Cooper Union, New York, whose various books have been so often reviewed in these pages. It is a journal that has held a prominent place in medical journalism for twelve years. Under the new management it is increased twenty per cent. in text; it is printed from entirely new type, and has a new design for the cover. Subscription $3.00 a year in advance.

